# Dr. Edward D. Clark

**Published:** 1915-03

**Authors:** 


**Dr. Edward D. Clark,** Buffalo, 1909, was found dead in his room in Syracuse, Feb. 13, from accidentally inhaling too much chloroform to relieve insomnia with which he suffered greatly. He was born in Buffalo, 27 years ago and received his education at the Masten Park High School. After receiving his medical degree, he served on the housestaff of the Buffalo General Hospital and then engaged in practice in Buffalo but, last fall, was appointed to the faculty of Syracuse University. He was a lieutenant in the medical corps of the National Guard, attached to the 65th Reg. of Buffalo and, recently, to the 3d at Syracuse. He was a member of the State and County Societies and of Erie Lodge, No. 161, F. & A. M. He was the only son of Dr. Edward Clark of Buffalo, Sanitary Supervisor of Erie, Niagara and Orleans Counties.

